# Myths and methodologies: Assessing glycaemic control and associated regulatory mechanisms in human physiology research

**DOI:** 10.1113/EP091433

**Published:** 2024-07-16

**Authors:** Elizabeth Wrench, Daren A. Subar, Theodoros M. Bampouras, Robert M. Lauder, Christopher J. Gaffney

**Affiliations:** ^1^ Lancaster Medical School, Health Innovation One, Sir John Fisher Drive Lancaster University Lancaster UK; ^2^ Royal Blackburn Hospital East Lancashire Hospitals NHS Trust Blackburn UK; ^3^ School of Sport and Exercise Sciences Liverpool John Moores University Liverpool UK

**Keywords:** glycaemia, glycaemic control, insulin sensitivity

## Abstract

Accurate measurements of glycaemic control and the underpinning regulatory mechanisms are vital in human physiology research. Glycaemic control is the maintenance of blood glucose concentrations within optimal levels and is governed by physiological variables including insulin sensitivity, glucose tolerance and β‐cell function. These can be measured with a plethora of methods, all with their own benefits and limitations. Deciding on the best method to use is challenging and depends on the specific research question(s). This review therefore discusses the theory and procedure, validity and reliability and any special considerations of a range common methods used to measure glycaemic control, insulin sensitivity, glucose tolerance and β‐cell function. Methods reviewed include glycosylated haemoglobin, continuous glucose monitors, the oral glucose tolerance test, mixed meal tolerance test, hyperinsulinaemic euglycaemic clamp, hyperglycaemic clamp, intravenous glucose tolerance test and indices derived from both fasting concentrations and the oral glucose tolerance test. This review aims to help direct understanding, assessment and decisions regarding which method to use based on specific physiology‐related research questions.

## 1 INTRODUCTION

Glycaemic control is the maintenance of blood glucose concentrations within optimal levels, and the measurement of glycaemic control is typically used within clinical environments for diagnostic purposes (Perlmuter et al., 2008). Maintaining glycaemic control helps to reduce the risk of secondary complications, making it an important clinical measure (Perlmuter et al., 2008). It can be measured from glycosylated haemoglobin (HbA1c), continuous glucose monitors (CGMs), finger‐prick blood glucose monitoring, oral glucose tolerance tests (OGTTs) and mixed meal tolerance tests (MMTTs) (American Diabetes Association Professional Practice Committee, 2022). Glycaemic control measurements do not, however, explain the physiology underlying the maintenance of euglycaemia or dysglycaemia. Physiological factors associated with glycaemic control include but are not limited to insulin sensitivity, β‐cell function and glucose tolerance.

Methods to measure glycaemic control are discussed, alongside methods to measure the associated physiology preceding abnormalities in glycaemic control. This includes methods to measure insulin sensitivity, glucose tolerance and β‐cell function. This review will consider the theory and procedure, the validity and reliability and any special considerations for each of the following methods: HbA1c, CGMs, OGTT, MMTT, hyperinsulinaemic euglycaemic clamp, hyperglycaemic clamp, intravenous glucose tolerance test (IVGTT) and indices derived from both fasting concentrations and the OGTT.

## 2 METHODS TO MEASURE GLYCAEMIC CONTROL

Glycaemic control, the maintenance of optimal blood glucose levels, is typically measured by HbA1c, regular blood glucose sampling, CGMs, OGTTs or MMTTs.

### 2.1 Glycosylated haemoglobin

#### 2.1.1 Theory and procedure

Glycosylated haemoglobin is often used as a measurement in clinical environments for diagnosis and prognosis and has previously been reviewed in detail for clinical populations (American Diabetes Association Professional Practice Committee, 2022). In research, it can be useful for measuring treatment effects and trends over time in epidemiological studies or for comparison between different populations (Nathan et al., 2007). Glycosylated haemoglobin is thought to be the gold standard for measuring glycaemic control and assessing outcomes in diabetes (Chehregosha et al., 2019). Haemoglobin has a lifespan of 120 days, and HbA1c occurs owing to the irreversible binding of glucose to haemoglobin (Nathan et al., 2007). Measurements of HbA1c therefore reflect mean blood glucose concentrations for the 8–12 weeks prior (Nathan et al., 2007). Glycosylated haemoglobin can be measured from a single blood sample via an assay (American Diabetes Association Professional Practice Committee, 2022).

#### 2.1.2 Validity and reliability

The logical validity of HbA1c is high because the irreversible binding of glucose to haemoglobin allows HbA1c to act as a cumulative measure of blood glucose concentration for the preceding 8–12 weeks (Chehregosha et al., 2019). Owing to the representation of mean blood glucose concentration over the period, variability is reduced in comparison to fasting plasma glucose (Owora, 2018). At the current diagnosis threshold for type 2 diabetes (≥6.5%, 48 mmol/mol), HbA1c has shown poorer sensitivity and higher specificity for discriminating type 2 diabetes for individuals previously undiagnosed, with 60% of individuals remaining undiagnosed when compared with OGTT diagnosis (Kaur et al., 2020; Pajunen et al., 2011). Glycosylated haemoglobin is a strong predictor of outcomes when measured close to diagnosis (Laiteerapong et al., 2019). Evidence suggests that HbA1c has poor reproducibility (intraclass correlation coefficient = 0.35) in normoglycaemic individuals (Simon et al., 1999).

#### 2.1.3 Special considerations

Glycosylated haemoglobin cannot measure glycaemic variability or acute glycaemic events, which are often correlated with symptoms from diabetes (American Diabetes Association Professional Practice Committee, 2022). The accuracy of the HbA1c measurement depends on the accuracy of the assay used, with a number of assays certified (American Diabetes Association Professional Practice Committee, 2022). Consideration needs to be taken for individuals who might be anaemic or have other diseases associated with a loss of erythrocytes or an inability of haemoglobin to bind to glucose (American Diabetes Association Professional Practice Committee, 2022). Differences in the mean age of red blood cells contributes to variability between HbA1c measures (Cohen et al., 2008). Glycosylated haemoglobin can also increase with age in normoglycaemia and can differ between ethnic populations, and therefore comparison between different age groups and ethnic populations requires additional consideration (Owora, 2018).

### 2.2 Continuous glucose monitoring

#### 2.2.1 Theory and procedure

**FIGURE 1:**
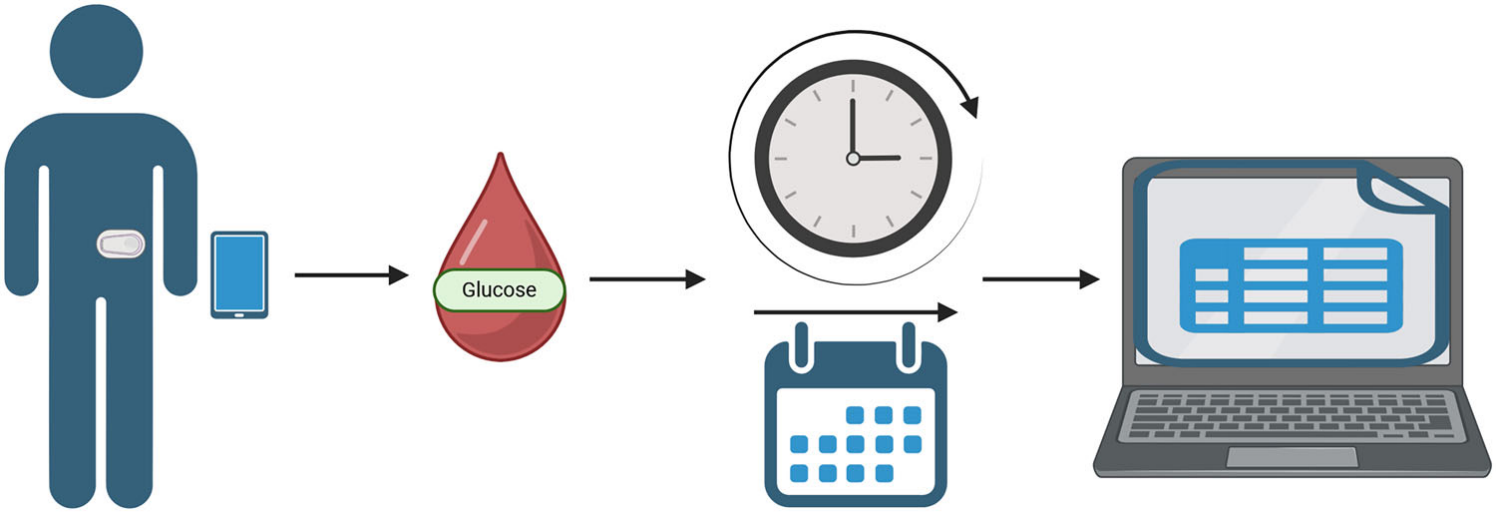
A continuous glucose monitor used within a research setting. The continuous glucose monitor is fitted to a participant on the lateral abdomen or posterior upper arm. Recordings are stored on the receiver device. Once the research period concludes, the data are exported from the receiver for collation in Excel or similar.

Continuous glucose monitors (CGMs), as shown in Figure 1, measure glucose concentrations from interstitial fluid using electrochemical technology to assess glycaemic control (Davison et al., 2022). Continuous glucose monitors allow ‘free‐living’ glycaemia to be recorded throughout the day and night (Lee et al., 2021). Measurements are recorded every 1–15 min and are stored immediately on the receiver or mobile application for later extraction and processing (Bergenstal, 2018). In addition to mean glucose, calculations can also be carried out to provide additional insight into overall glycaemic control, such as glycaemic variability and the amplitude of glycaemic variability, the J‐index (based on the mean and SD of all glucose values), glucose management indicator and time in the range of 3.9–10 mmol/L (70–180 mg/day) (Bergenstal, 2018).

#### 2.2.2 Validity and reliability

The logical validity of CGMs for measuring glycaemic control is high, with blood glucose concentration measured at regular intervals. Glucose measurements are, however, sampled from interstitial fluid, which results in a physiological delay versus circulatory glucose concentrations (Sinha et al., 2017). Average lag time is reported as 5–6 min in healthy adults but has decreased in newer models with lag times as low as ∼2 min (Alva et al., 2023; Sinha et al., 2017).

Continuous glucose monitors in normoglycaemic individuals show agreement with venous samples, but the accuracy of calculated measures of glycaemia and glycaemic variability deviated significantly, overestimating glycaemia during the day and underestimating glycaemic variability (Akintola et al., 2015). Accuracy of CGMs is acceptable for non‐critically ill and critically ill inpatients, paediatric patients (4‐ to 5‐year‐olds) and adults with type 1 and type 2 diabetes, with accuracy highest when glycaemic control is stable (Alva et al., 2023; Finn et al., 2023; Lindner et al., 2021). A recent meta‐analysis, however, found poor accuracy for detection of hypoglycaemia, and therefore care should be taken when CGMs are used in research where the detection of hypoglycaemia is important (Lindner et al., 2021). For measures of overall glycaemic control from CGMs, an average blood glucose concentration of >26 days has shown to correlate best with HbA1c (Tozzo et al., 2024).

Bland–Altman analyses have shown that CGMs underestimate the postprandial rise in glucose concentration for healthy individuals but overestimate plasma glucose during steady‐state exercise, specifically in women (Barua et al., 2022; Herrington et al., 2012). For accurate measurements of blood glucose concentration in these conditions, finger‐prick blood sampling might be superior. In a comparison of two of the most popular CGM brands, Abbott and Dexcom, within‐person and between‐sensor variation was high in individuals with type 2 diabetes over a 3‐month period, suggesting poor long‐term reliability (Selvin et al., 2023). This might be attributable to biological variation and differences in sensor technology (Selvin et al., 2023). Inter‐day variations are also poor for normoglycaemia, prediabetes and diabetes (Matabuena et al., 2023). Individuals with type 2 diabetes show the least variation, thought to be owing to poor adaption to functional changes (Matabuena et al., 2023). Further research is required on the reproducibility of CGMs.

#### 2.2.3 Special considerations

Continuous glucose monitors are useful for therapeutic use, determining the effect of an intervention on glycaemic control, and are less invasive than regular finger‐prick blood samples. In research, it is recommended to calibrate CGMs with finger‐prick samples. Fitting requires a brief ∼10 min visit to a laboratory, and participant burden is relatively low. Participants are often required to wear the CGM for a long period (typically, 24 h to 2 weeks) to provide an accurate representation of glycaemic control, and this increases participant burden.

Medications and supplements, such as paracetamol and ascorbic acid (vitamin C), can interfere with the electrochemistry of CGMs and therefore must be controlled appropriately (Heinemann, 2022). Cost and lifespan vary between brands, but systems typically require a sensor, transmitter and receiving device (or app).

Investigations into the impact of visceral adiposity on the accuracy of CGM readings are limited, but no association was observed between participant characteristics (body mass index, sex, and mean age) and pooled sensitivity and specificity in a meta‐analysis (Lindner et al., 2021). No differences were also found between body composition or the location of sensor insertion (arm vs. abdomen) on device accuracy (Abraham et al., 2023; Steineck et al., 2019).

### 2.3 Oral glucose tolerance test

#### 2.3.1 Theory and procedure

**FIGURE 2:**
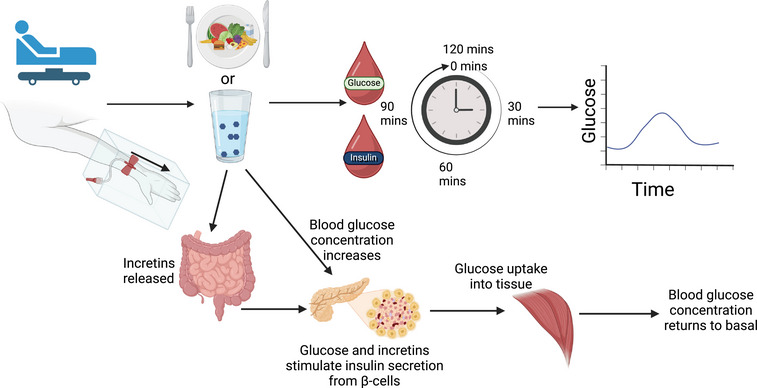
A summary of an oral glucose tolerance test (OGTT) or a mixed meal tolerance test (MMTT). The participant is seated in a comfortable semi‐supine position, with their hand placed in a heated box. After 15 min, a retrograde cannula is placed in the dorsal surface of their hand, and a fasting blood sample is taken. The participant then consumes a glucose load (75 g dextrose in 300 mL water) for an OGTT or a standardised meal for a MMTT, and blood samples are taken regularly. From each of these samples, glucose is usually measured immediately, with plasma and serum extracted for later determination of insulin and any other analytes. A response curve is plotted with the concentration at each time point.

An OGTT, as shown in Figure 2, assesses the ability of an individual to process a large glucose load (Jagannathan et al., 2020). Oral glucose tolerance tests are used clinically to diagnose glucose intolerance or in research settings to assess glucose handling, insulin sensitivity and β‐cell function, both typically estimated from indices, as shown in table 1 (Hannon et al., 2018; Muniyappa et al., 2008). Following an overnight fast, for a standard clinical OGTT, participants consume a glucose load (75 g dextrose in 300 mL water), with blood samples taken every 30 min for the subsequent 2 h (Stumvoll et al., 2000). Variations of the test during research, however, include different glucose doses (50–100 g), different sampling periods and different administration methods (Jagannathan et al., 2020). Blood glucose concentrations can be analysed immediately or processed and stored for analysis along with insulin at a later date, typically via an enzyme‐linked immunosorbent assay or radioimmunoassay (Matsuda & DeFronzo, 1999). Glucose and insulin concentrations can be plotted at each time point, producing a curve to understand an individual's glycaemic control, glucose tolerance and insulin sensitivity (Jagannathan et al., 2020).

#### 2.3.2 Validity and reliability

The OGTT activates a physiological response to a glycaemic load. This is more representative of continuously changing glycaemia and the negative feedback mechanisms between glucose and insulin postprandially (Otten et al., 2014). Time to peak glucose represents the ability of β‐cells to secrete sufficient insulin quickly, whereas 2 h glucose concentrations represent the action of insulin on glucose uptake to return to basal (Chung et al., 2017). Development of changes to postprandial glycaemic control typically occurs before changes in fasting blood glucose concentration (Jagannathan et al., 2020). The OGTT can therefore detect dysglycaemia more effectively than fasting measures (Jagannathan et al., 2020). Direct measures of an individual's glucose tolerance and glycaemic control can be made, but whole‐body insulin sensitivity has to be estimated via insulin sensitivity indices (Otten et al., 2014).

The OGTT can differentiate effectively between impaired glucose tolerance, diabetes and normal glucose tolerance when 2 h post‐glucose values are compared, and therefore indicates good construct validity (Bartoli et al., 2011). Test–retest reliability can be poor, particularly in individuals with impaired glucose metabolism (Gordon et al., 2011; Ko et al., 1998). Reproducibility can be improved by following standardised protocols and by ensuring careful handling and analyses of samples (Ko et al., 1998). Potential intra‐ and inter‐individual variability in OGTTs can be dictated by glucose absorption and the incretin response, and therefore reproducibility needs to be considered (Hücking et al., 2008).

#### 2.3.3 Special considerations

The OGTT is less invasive, time consuming and complex, reducing participant burden and increasing simplicity in comparison to glycaemic clamp methodologies and IVGTTs, discussed below. Glucose tolerance is tested in relatively comparable real‐world physiological conditions. This allows for measurement of dynamic changes in glucose and insulin concentrations (Hücking et al., 2008). Any samples obtained for analysis at a later date should be stored at ∼≤−80°C to prevent degradation of analytes (Kong et al., 2017).

Oral glucose tolerance test methodologies differ, especially between those used in clinical and research settings. Evidence on the differences between using arterialised venous versus venous blood sampling to measure metabolites has been documented (Edinburgh et al., 2017). To allow for the less invasive collection of arterialised distal blood samples, participants can place their hand in a heated box (∼41°C; Tam et al., 2012) for ∼15 min prior to samples being taken and between sampling, to allow for arterialisation of the blood via arterial–venous shunting (Brooks et al., 1989). When comparing arterial venous and venous samples, arterialised venous blood samples (achieved by heating the hand to ∼37°C) have shown to provide metabolite concentrations that are better estimates of arterial samples (Edinburgh et al., 2017).

Evidence on the impact of retrograde versus anterograde cannulation on differences in metabolites measured from either arterialised venous or venous blood samples is limited (McNair et al., 1995; Rowe et al., 1994). Retrograde cannulation increases the rates of cannulation failure, is reported to be more painful by participants, and when compared, anterograde versus retrograde cannulation did not alter the reproducibility of measurements taken from intravenous glucose tests (McNair et al., 1995; Rowe et al., 1994). To allow for comparisons between studies, essential reporting of the methods used is important, but there is still no clear consensus of the specific method to be adopted. This is likely to depend on the population to be studied (e.g., retrograde cannulation is not recommended for children and other vulnerable populations) and the availability of specialist staff or equipment (Edinburgh et al., 2017).

### 2.4 Mixed meal tolerance test

#### 2.4.1 Theory and procedure

A MMTT, as shown in Figure 2, assesses the ability of an individual to process a meal (Brodovicz et al., 2011). This method has the greatest ecological validity, being representative of daily life and the physiological processing of glucose. The methodology is similar to an OGTT but assesses the impact of proteins and fat alongside glucose on glycaemic control, β‐cell function, glucose tolerance and insulin sensitivity (Brodovicz et al., 2011). Proteins, fat and glucose all stimulate the incretin response involved in insulin secretion (Brodovicz et al., 2011). Differences have therefore been found in the β‐cell function and the insulin and glucose concentrations determined between an OGTT and a MMTT (Brodovicz et al., 2011). The meal has not been standardised between studies but typically includes carbohydrates, fat and protein; evidence of meals are provided in the following studies: Brodovicz et al. (2011); Rijkelijkhuizen et al. (2009); and Shankar et al. (2016). Samples are taken at regular time points for ≤5 h (Shankar et al., 2016).

The incremental area under the curve can be calculated to determine C‐peptide, insulin and glucose responses (Kössler et al., 2021). β‐Cell function can be estimated from insulin or often, owing to its secretion in equimolar concentrations and limited hepatic clearance, C‐peptide (Brodovicz et al., 2011). Indices to measure β‐cell function include the insulinogenic index and the ratio of insulin to glucose area under the curve (Brodovicz et al., 2011; Shankar et al., 2016). Insulin sensitivity can be determined from insulin sensitivity indices, such as Matsuda and the Oral Glucose Insulin Sensitivity (OGIS) index (Brodovicz et al., 2011; Rijkelijkhuizen et al., 2009).

#### 2.4.2 Validity and reliability

A MMTT is the most ecologically valid method for assessing glycaemic control, the effectiveness of β‐cell secretion and for estimating insulin sensitivity because it replicates the daily postprandial response (Brodovicz et al., 2011).

The MMTT is able to discriminate differences in both β‐cell function and insulin sensitivity across the metabolic spectrum from normal glucose tolerance to prediabetes and diabetes (Shankar et al., 2016). Moderate reproducibility of the MMTT has been reported, with reproducibility ranging from weak to strong in different populations, with the test being weakly reproducible in individuals with type 2 diabetes (Shankar et al., 2016). Intra‐individual coefficients of variation are comparable when liquid meals differing in nutritional content are compared (Kössler et al., 2021). Estimates of β‐cell function are higher in a MMTT than in an OGTT, thought to be explained by increased β‐cell secretion during the MMTT (Rijkelijkhuizen et al., 2009).

Equations such as area under the curve, Matsuda and Stumvoll methodologies, discussed in Table 1, can estimate insulin sensitivity from the MMTT (Rijkelijkhuizen et al., 2009). The correlation between MMTT‐ and OGTT‐derived indices is high (Rijkelijkhuizen et al., 2009). Frequently compared with the OGTT and associated indices, further research is required on the agreement of the MMTT with the gold‐standard hyperinsulinaemic euglycaemic and hyperglycaemic clamps.

**TABLE 1:** A summary of oral glucose tolerance test‐derived indices.

Index	Equation
Matsuda (Matsuda & DeFronzo, 1999)	=$\frac{10,000}{\sqrt{\left(\mathrm{Glucos}e_{0\;\min}\times \mathrm{Insuli}n_{0\;\min}\right)\times \left(\mathrm{Glucos}e_{\mathrm{mean}}\times \mathrm{Insuli}n_{\mathrm{mean}}\right)}}$
Cederholm (Cederholm & Wibell, 1990)	= $\frac{\frac{\frac{\mathrm{Glucose}\;\mathrm{load}(\mathrm{mg})}{120}+\left(\mathrm{Glucos}e_{0\;\min}\;-\;\mathrm{Glucos}e_{120\;\min}\right)\;\times \;1.15\;\times \;180\;\times \;0.19\;\times \;\frac{\mathrm{Body}\;\mathrm{mass}}{120}}{\mathrm{Glucos}e_{\mathrm{mean}}}}{\log(\mathrm{Insuli}n_{\mathrm{mean}})}$
Gutt (Gutt et al., 2000)	$=\frac{\frac{\mathrm{Glucose}\;\mathrm{load}\;\left(\mathrm{mg}\right)+\left(\mathrm{Glucos}e_{0\;\min}-\mathrm{Glucos}e_{120\;\min}\right)\times \;0.19\times \;\frac{\mathrm{Body}\;\mathrm{mass}}{120}}{\mathrm{Glucos}e_{\mathrm{mean}(0,120\;\min)}}}{\log(\mathrm{Insuli}n_{\mathrm{mean}(0,120\;\min)})}$
Stumvoll ISI (Stumvoll et al., 2000)	$=0.157-4.576\;\times \;10^{-5}\times \;\mathrm{Insuli}n_{120\;\min}-$0.00519× $\mathrm{Glucos}e_{90\;\min}-$0.000299 × $\mathrm{Insuli}n_{0\;\min}$
Stumvoll ISI* (Stumvoll et al., 2000)	$=0.226-0.0032\;\times \;\mathrm{body}\;\mathrm{mass}\;\mathrm{index}\left(\frac{\mathrm{kg}}{m^{2}}\right)-0.0000645\;\times \;\mathrm{Insuli}n_{120\;\min}-0.00375\;\times \;\mathrm{Glucos}e_{90\;\min}$
OGIS (Mari, Pacini, et al., 2001)	A complex computation including the following variables: glucose concentration (0, 90, 120 min), insulin concentration (0, 90 min), glucose dose (in grams), body mass and height. The calculation can be programmed on a spreadsheet or online

#### 2.4.3 Special considerations

The MMTT has similar considerations to the OGTT. The test is less invasive and easier to perform than the gold‐standard measures of insulin sensitivity and β‐cell function, but is less controlled and cannot determine insulin sensitivity directly. A standardised test meal is not used consistently within research. Some use a liquid meal; others use a solid meal or a combination of both, and the composition of branded nutritional meals is likely to change over time (Brodovicz et al., 2011; Shankar et al., 2016). The MMTT typically lasts ∼4 h, with samples taken approximately every 30 min, but can vary (Brodovicz et al., 2011). Evidence on the validity and reliability of the MMTT in different ethnic groups is limited (Ladwa et al., 2021).

## 3 METHODS TO MEASURE THE PHYSIOLOGY UNDERPINNING GLYCAEMIC CONTROL

Impairments in insulin sensitivity, β‐cell secretion and glucose tolerance occur significantly earlier than changes in glycaemic control (Kahn et al., 2014). Therefore, effective measurements of factors underpinning glycaemic control are important in physiological research for the understanding, prevention and intervention of associated diseases.

Insulin sensitivity is the effective metabolic action of the hormone insulin (Katz et al., 2000). The more insulin sensitive an individual is, the more effective their body is at physiologically disposing of glucose into tissue (Bird & Hawley, 2017). In clinical populations, impaired insulin sensitivity contributes to abnormal glycaemic control owing to reduced whole‐body glucose uptake (Bird & Hawley, 2017). Insulin sensitivity can be measured directly by the hyperinsulinaemic euglycaemic clamp, which is the gold standard for measuring tissue insulin sensitivity (DeFronzo et al., 1979). Insulin sensitivity can also be estimated from the hyperglycaemic clamp, minimal model of the IVGTT, insulin sensitivity indices calculated from the OGTT, MMTT and fasting glucose and insulin concentrations.

Glucose tolerance is the ability to return to euglycaemic concentrations after a perturbation (Ahrén, 2013). Impaired glucose tolerance, owing to poor glucose disposal, can result in blood glucose concentrations remaining outside of euglycaemic levels for a prolonged period of time, and this can contribute to abnormal glycaemic control observed in prediabetes (Ahrén, 2013). Glucose tolerance can be measured from an IVGTT, an OGTT or a MMTT. Glucose tolerance tests, typically the OGTT, can be used for diagnosis of type 2 diabetes in clinical settings. Within research, these methods can be used to understand glucose tolerance directly and other factors indirectly, such as insulin sensitivity (Muniyappa et al., 2008).

β‐Cell function results from β‐cell sensitivity to glucose, insulin secretion and the effects of incretin hormones, requiring β‐cells to effectively produce, store and secrete insulin to ensure that euglycaemia is maintained (Hannon et al., 2018). Impairments in β‐cell function reduce the effectiveness of insulin secretion, resulting in hyperglycaemia. The hyperglycaemic clamp is the gold standard for the assessment of β‐cell sensitivity to glucose (Hannon et al., 2018). The OGTT, IVGTT and MMTT can also be used to assess β‐cell function (Hannon et al., 2018). Alongside an assessment of β‐cell function, a measure of insulin sensitivity needs to be incorporated to account for the hyperbolic relationship between insulin sensitivity and β‐cell secretion (Hannon et al., 2018; Kahn, 2003). Both β‐cell dysfunction and decreased insulin sensitivity precede hyperglycaemia, which can be measured from glycaemic control methods (Kahn, 2003).

### 3.1 Hyperinsulinaemic euglycaemic clamp

#### 3.1.1 Theory and procedure

**FIGURE 3:**
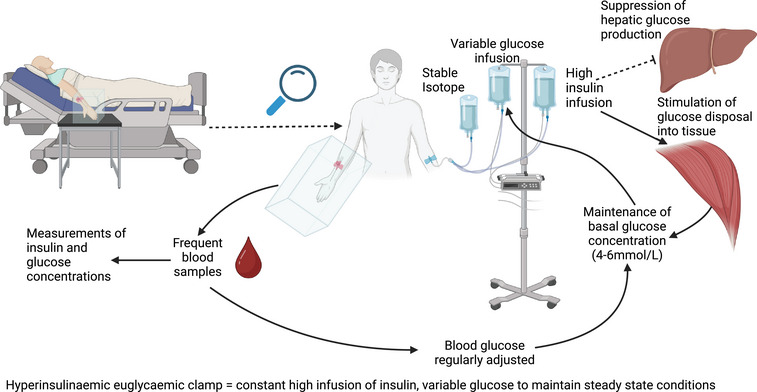
Hyperinsulinaemic euglycaemic clamp. A participant is seated in a semi‐supine position, and their hand is placed in a heated box (∼41°C; Tam et al., 2012). On the opposite arm, insulin is infused at a high concentration along with glucose at a variable rate to maintain a stable glucose concentration (and a stable isotope if glucose uptake is to be traced). A cannula is inserted into a peripheral wrist vein, and the lower arm is placed in a heated box (if arterialised samples are required), and frequent blood samples are taken every 2–5 min. The glucose concentration is analysed immediately to inform glucose infusion adjustments. Insulin concentrations can be determined later.

Hyperinsulinaemic euglycaemic clamps, as shown in Figure 3, are the gold standard for estimating tissue insulin sensitivity and are reviewed extensively elsewhere (DeFronzo et al., 1979; Heise et al., 2016; Uwaifo et al., 2002). In brief, the hyperinsulinaemic euglycaemic clamp involves the infusion of insulin to increase and maintain high plasma insulin concentrations, traditionally ∼100 mIU/mL (DeFronzo et al., 1979). To reach the desired hyperinsulinaemic concentrations, a priming dose acutely raises plasma insulin concentrations (Picchini et al., 2005). Glucose concentration is held at basal levels (4–6 mmol/L; Davison et al., 2022) by an additional variable glucose infusion, preventing hypoglycaemia (DeFronzo et al., 1979). The high insulin concentration aims to suppress hepatic glucose production completely, meaning that the only glucose available is from the exogenous supply. The glucose infusion rate required to maintain basal glucose concentrations is therefore representative of glucose disposal into tissue (DeFronzo et al., 1979). To estimate insulin sensitivity, the glucose disposal rate is typically normalised by body weight or fat‐free mass (Muniyappa et al., 2008).

The hyperinsulinaemic euglycaemic clamp can also be performed at different insulin doses in a single test (Sowell et al., 2003). The insulin infusion starts at the lowest dose, then increases to a higher dose at a specific time point (Sowell et al., 2003). A lower insulin infusion dose helps to determine insulin sensitivity, whereas a higher insulin infusion dose can be useful to determine the maximal responsiveness of an individual to insulin (Sowell et al., 2003).

#### 3.1.2 Validity and reliability

The logical validity of this test is high as long as hepatic glucose production is sufficiently suppressed by the continuous high‐dose insulin infusion (Tam et al., 2012). The variable glucose infusion rate to maintain basal concentrations therefore represents glucose uptake and utilisation reflective of insulin sensitivity (Tam et al., 2012). Hyperinsulinaemic euglycaemic clamps create highly standardised environments in which differences in individuals can be detected with the highest sensitivity, rather than replicating real‐life physiological conditions. This, however, results in limited ecological validity (Heise et al., 2016; Hücking et al., 2008).

The hyperinsulinaemic euglycaemic clamp can differentiate successfully between normoglycaemic individuals and those with diabetes, and definitions of cut‐off points for insulin resistance have been described previously (Tam et al., 2012). The clamp has also been shown to differentiate between obese and non‐obese individuals, independent of age, indicated by reduced glucose infusion rates (Karakelides et al., 2010).

The clamp is repeatable over both a shorter period (3–4 weeks) and a longer period (∼2.30 years) in healthy adults (DeFronzo et al., 1979; James et al., 2020). Based on methods suggested by Bland and Altman, the intra‐individual differences lay within the 95% limits of agreement and were smaller than the repeatability coefficient (±0.025), confirming the reproducibility of the test over the longer period (James et al., 2020).

### 3.2 Hyperglycaemic clamp

#### 3.2.1 Theory and procedure

**FIGURE 4:**
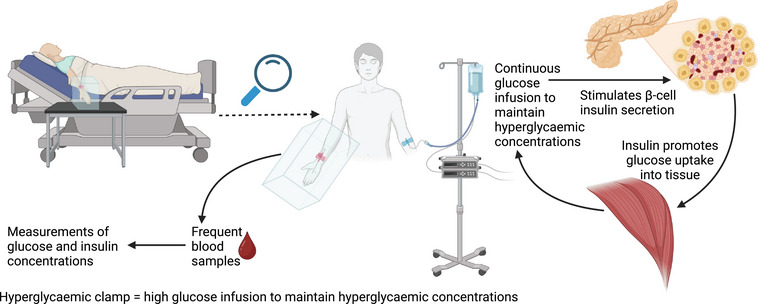
Hyperglycaemic clamp. A participant is seated in a semi‐supine position, and their hand is placed in a heated box (∼41°C; Tam et al., 2012). On the opposite arm, for a hyperglycaemic clamp, glucose is infused intravenously to maintain high glucose concentrations (along with a stable isotope if glucose uptake is to be traced). A cannula is inserted into a peripheral wrist vein, and the lower arm is placed in a heated box (if arterialised samples are required), and frequent blood samples are taken every 2–5 min. The glucose concentration is analysed immediately to inform glucose infusion adjustments. Insulin concentrations can be determined later.

Hyperglycaemic clamps, as shown in Figure 4, are the gold‐standard method for estimating the function of β‐cells (DeFronzo et al., 1979; Elahi, 1996; Uwaifo et al., 2002). Estimations of insulin sensitivity, glucose effectiveness and insulin clearance can also be made (Uwaifo et al., 2002). Participants are infused with a variable glucose concentration to maintain high plasma glucose concentrations [typically > ∼6.9 mmol/L (125 mg/dL); DeFronzo et al., 1979]. The aim of the high plasma glucose concentration is to activate insulin secretion, which allows β‐cell function to be assessed (DeFronzo et al., 1979).

In individuals with impaired glucose tolerance and decreased insulin sensitivity, impairments of insulin secretion in the first‐phase response can be detected in the early stages of the disease (Hannon et al., 2018). The hyperglycaemic clamp allows independent assessment of first‐ and second‐phase insulin secretion to give a better understanding of the underlying physiology (DeFronzo et al., 1979). Tissue insulin sensitivity can also be estimated from the hyperglycaemic clamp, using the ratio of glucose metabolism to plasma insulin concentration or insulin sensitivity indices, for example (DeFronzo et al., 1979; Elahi, 1996; Mitrakou et al., 1992).

#### 3.2.2 Validity and reliability

Hyperglycaemic clamps have high logical validity, aiming to stimulate and maintain a β‐cell response by infusing a high concentration of glucose throughout the test (DeFronzo et al., 1979; Meneilly & Elliott, 1998). When the same hyperglycaemic concentration is maintained, β‐cell responses can be compared between populations (DeFronzo et al., 1979; Meneilly & Elliott, 1998). The hyperglycaemic clamp has limited ecological validity owing to the supraphysiological levels of glucose infused over a long period that do not represent daily life (Hücking et al., 2008).

The hyperglycaemic clamp can accurately and reliably differentiate measures of β‐cell function, insulin sensitivity and insulin clearance between individuals at different stages of the pathophysiological progression from normal glucose tolerance to impaired glucose tolerance and type 2 diabetes, along with youth and adult populations, and with a range of obesity (Hannon et al., 2018; Mather et al., 2021; Meneilly & Elliott, 1998). Test–retest reliability was high over a period of 3–4 weeks (DeFronzo et al., 1979).

Estimations of insulin sensitivity from the hyperglycaemic clamp have shown to correlate with direct measures of tissue sensitivity from the gold‐standard hyperinsulinaemic euglycaemic clamp (DeFronzo et al., 1979; Mitrakou et al., 1992). In children, the two clamps were significantly correlated for measures of insulin sensitivity, but assumptions regarding equivalence could not be made (Uwaifo et al., 2002).

#### 3.2.3 Special considerations of glycaemic clamps

Despite glycaemic clamps being the gold‐standard method, the complexity of the methods, the availability of equipment and clinically trained staff support and the cost of equipment make the methods logistically and practically challenging. Glycaemic clamps have a high participant burden owing to the invasive nature, period of fasting prior (∼12 h) and time taken for the test to be carried out (≥3 h) (DeFronzo et al., 1979; Tam et al., 2012). This makes them challenging to use in vulnerable or high‐risk populations, including children and adolescents, and they are never used for clinical purposes, only for research.

Careful consideration needs to be given to determine the concentration and speed of infusate in order that blood insulin and glucose levels do not significantly increase or decrease to harmful concentrations (DeFronzo et al., 1979). In hyperinsulinaemic euglycaemic clamps, isotopic or radioactive tracers can be used to monitor the level of hepatic glucose production to ensure that endogenous glucose production is completely suppressed (Heise et al., 2016). Mathematical methods to determine the contribution of endogenous glucose to glucose uptake by using tracers are discussed elsewhere (Finegood et al., 1987). Specific tracers can also provide additional evidence during clamps on metabolic pathways and the metabolic fate of a range of molecules, including glucose, fat and protein metabolism (Brook & Wilkinson, 2020).

The aim of clamp methodologies is to create highly standardised environments, in which differences in individuals can be detected with the highest sensitivity, rather than replicating real‐life physiological conditions (Heise et al., 2016). The clamp therefore does not take into consideration the dynamic relationship between insulin and glucose under normal physiological conditions (Heise et al., 2016).

Hyperinsulinaemic euglycaemic and hyperglycaemic clamps are the most common examples of glycaemic clamps, but other clamps are available to investigate different research questions, including hyperinsulinaemic hypoglycaemic clamps, isoglycaemic clamps and hyperinsulinaemic hyperglycaemic clamps, among others (Fabricius et al., 2021; MacLaren et al., 2011).

### 3.3 Intravenous glucose tolerance test

#### 3.3.1 Theory and procedure

**FIGURE 5:**
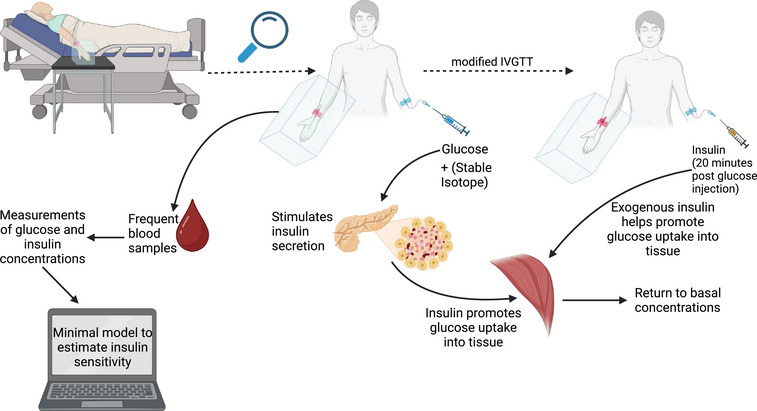
A summary of an intravenous glucose tolerance test (IGVTT). The participant is seated in a comfortable semi‐supine position, with their hand placed in a heated box. After 15 min, a retrograde cannula is placed in a peripheral wrist vein, and a fasting blood sample is taken. The participant is then injected with a glucose load, and blood samples are taken at regular intervals; a tracer can also be injected at this time point. For a modified IVGTT, an insulin dose is injected 20 min after the glucose load. From each of these samples, glucose is usually measured immediately, with plasma and serum extracted for later determination of insulin and any other analytes. The minimal model can then be used to estimate insulin sensitivity from the insulin and glucose concentrations.

The IVGTT, as shown in Figure 5, allows glucose tolerance, β‐cell function and insulin sensitivity to be estimated from a single test (Bergman, 2021; Bergman et al., 1979; Godsland et al., 2024). An IVGTT involves an intravenous glucose dose, typically 0.3, 0.5 or 1 g/kg body weight as a 20%–50% glucose solution, injected over 1–3 min (Ahrén, 2013; Godsland et al., 2024). Both glucose and insulin plasma concentrations are sampled frequently postinfusion (typically, −10 min, −1 min, then for the first 30 min at 2–5 min intervals, 30–60 min at 5–10 min intervals, and >60 min at 30 min intervals; Ahrén, 2013; Bergman, 2021). The test directly measures glucose tolerance, which is how effectively an individual processes the glucose infusion to return to fasting concentrations (Bergman et al., 1979).

β‐Cell secretion can be estimated from the 10‐min period post‐glucose infusion (acute insulin response to glucose) (Godsland et al., 2024). C‐Peptide concentrations can also be measured to understand β‐cell secretion during an IVGTT (Hannon et al., 2018). C‐Peptide is secreted in equimolar concentrations to insulin but is not degraded by hepatic systems and can therefore reflect a more accurate measure of insulin secretion rates (Hannon et al., 2018).

Insulin sensitivity can be estimated from the IVGTT (Bergman, 2021; Bergman et al., 1979). The minimal model is most commonly used which estimates both glucose effectiveness (glucose kinetics at fasting insulin concentrations) and insulin sensitivity (the role of insulin in glucose kinetics) (Bergman, 2021; Bergman et al., 1979). The theory behind the minimal model links together the negative feedback loop of glucose and insulin into two separate subsystems, with insulin concentration as the input and glucose concentration as the output (Bergman et al., 1979). The modified IVGTT includes an infusion of, most commonly insulin, but also tolbutamide, 20 min post‐glucose injection to measure insulin sensitivity accurately in individuals with impaired insulin secretion (Bergman, 2021).

#### 3.3.2 Validity and reliability

The logic behind the IVGTT is valid because a measured dose of glucose is infused with an assessment of how the individual responds to the perturbation (Bergman, 2021). Glucose tolerance is determined from the time taken to respond to the glucose load and return to euglycaemia. β‐Cell function can be determined from the acute first‐phase insulin (or C‐peptide) response as the glucose load stimulates β‐cell secretion. The minimal model can estimate insulin sensitivity from the IVGTT.

The acute insulin response to glucose, determined from the Minimal Model and the IVGTT, and the hyperglycaemic clamp, were found to correlate significantly in healthy individuals (*P *< 0.005, *r *= 0.75; Hansen et al., 2020; Korytkowski et al., 1995). However, using the acute insulin response to glucose as a measure of β‐cell function in individuals with hyperglycaemia is limited owing to dysfunction in the acute insulin response (Hansen et al., 2020; Korytkowski et al., 1995). Inter‐individual variation is high for normoglycaemic individuals and for those with metabolic syndrome and type 2 diabetes (Bardet et al., 1989; Hansen et al., 2020). Test–retest reliability is high, determined from IVGTTs carried out 9 months apart (Bardet et al., 1989).

When estimating insulin sensitivity using the minimal model, the test discriminated decreasing insulin sensitivity associated with increasing body mass index (Bergman et al., 1987). However, the test was poorly correlated with insulin sensitivity for individuals with type 2 diabetes (*r* = 0.3, *P* = 0.085), with only ∼50% of insulin sensitivity estimations definitive (Saad et al., 1994). Evidence suggests that the simplicity of the minimal model underestimates insulin sensitivity and overestimates glucose effectiveness (Saad et al., 1994). Insulin sensitivity values indistinguishable from zero contribute to underestimations, particularly in individuals with diabetes, and allowing negative insulin sensitivity values has been suggested (Ni et al., 1997). A two‐compartment minimal model involving a tracer has also been suggested to increase accuracy (Toffolo & Cobelli, 2003). The minimal model as a measure of insulin sensitivity has found to be reproducible 3 weeks apart in normoglycaemic young males (Ferrari et al., 1991).

#### 3.3.3 Special considerations

The IVGTT is simpler to perform than the gold‐standard hyperinsulinaemic euglycaemic clamp but is still highly invasive, with a high participant burden. Although the method can be used in vulnerable populations, such as women during pregnancy and children, the test can be challenging, with mild adverse events (Skajaa et al., 2020; Tompkins et al., 2010). Indeed, modifications to the protocol might increase safety and comfort. IVGTTs have previously been used in large epidemiological studies, such as the Insulin Resistance Atherosclerosis Study (IRAS), but require large capacity, funding and expertise to be carried out (Muniyappa et al., 2008; Wagenknecht et al., 1995). Although the insulin sensitivity of individuals of different ethnicities has been compared using the IVGTT (Ellis et al., 2012), evidence on the reliability of using the IVGTT in different ethnic populations is limited.

To measure only the impact of insulin on glucose disposal, particularly for insulin sensitivity, stable isotopes can be injected intravenously to improve the precision of the model (Toffolo & Cobelli, 2003). The use of labelled isotopes also allows for a two‐compartment rather than a one‐compartment model to estimate insulin sensitivity (Toffolo & Cobelli, 2003).

Insulin sensitivity must be measured and taken into account to measure β‐cell function accurately; this is because of the tight relationship between insulin secretion and insulin action (Hannon et al., 2018). The disposition index, discussed in detail elsewhere, describes the β‐cell sensitivity–secretion relationship (Bergman et al., 2002).

### 3.4 Oral glucose tolerance test‐derived indices

#### 3.4.1 Theory and procedure

Both insulin release and insulin sensitivity are interdependent and provide useful information on glucose homeostasis. Insulin sensitivity cannot be determined directly from the glucose and insulin concentrations of an OGTT (Stumvoll et al., 2000). Table 1 highlights some of the indices that assess insulin sensitivity from concentrations measured during the OGTT.

#### 3.4.2 Validity and reliability

Oral glucose tolerance test‐derived indices are developed based on the feedback mechanism of insulin and glucose to allow for an estimation of insulin sensitivity. They typically use both glucose and insulin concentrations at specific time points during the OGTT, with some indices including additional variables (Hudak et al., 2021; Otten et al., 2014).

Oral glucose tolerance test‐derived indices have a higher discriminant ratio [1.92 (1.59–2.33)] to determine metabolic differences than indices derived from fasting concentrations [1.82 (1.51–2.22)], but poorer reproducibility (Hudak et al., 2021). The Matsuda index and OGIS both show good agreement, based on Bland–Altman analysis, and the best correlation with the hyperinsulinaemic euglycaemic clamp, with OGIS found to have the best test–retest reliability and the Matsuda index found to have the worst (Hudak et al., 2021; Leonetti et al., 2004). Evidence within the literature suggests that the Cederholm index has the poorest correlation with the hyperinsulinaemic euglycaemic clamp (Hudak et al., 2021; Otten et al., 2014). The increased number of variables included in the equation could lead to increased variability (Hudak et al., 2021).

#### 3.4.3 Special considerations

The reproducibility of the indices is impacted directly by the reproducibility and quality of the OGTT carried out, and therefore the OGTT should be highly controlled.

Care should be taken when comparing mixed‐race or mixed‐sex populations using insulin sensitivity indices (Pisprasert et al., 2013). For example, estimation using indices has been shown to predict higher insulin resistance for African American populations than European Americans even though measurements by the hyperinsulinaemic euglycaemic clamp were similar, probably owing to differences in the physiological mechanisms behind insulin sensitivity that the indices are based on (Pisprasert et al., 2013). Out of the indices discussed in this review, the Matsuda index was found to be the most reliable measure of insulin sensitivity in African Americans (Pisprasert et al., 2013). The Matsuda index was also found to be a valid measure of insulin sensitivity in South Asians (Trikudanathan et al., 2013).

The indices use slightly different variables to estimate insulin sensitivity. The Matsuda index is a simple equation, using both fasting and mean insulin and glucose concentrations to measure insulin sensitivity, but does not consider any demographic factors, such as body mass or glucose distribution volume, which could impact the insulin sensitivity determined (Matsuda & DeFronzo, 1999). The Cederholm index uses four time points during the OGTT and takes into consideration the body mass of an individual, but the number of variables included is thought to impact its correlation with clamp measures (Cederholm & Wibell, 1990). Gutt built upon the equation by Cederholm and Wibell (1990), reducing the number of variables and increasing correlation with the hyperinsulinaemic clamp (Gutt et al., 2000; Otten et al., 2014). Stumvoll used linear regression to determine which variables are the best predictors of insulin sensitivity determined by the hyperinsulinaemic clamp, producing an equation with body mass index (ISI*) and one without (ISI) (Stumvoll et al., 2000). OGIS is the most complex equation, using unknown predictor variables determined from a comparison of an OGTT and hyperinsulinaemic clamp, along with height, body weight, glucose dose and 0, 90 and 120 min glucose and insulin concentrations (Mari, Pacini, et al., 2001). It has shown good agreement and reproducibility with the hyperinsulinaemic clamp, and online software is available to assist with computation (Hudak et al., 2021; Leonetti et al., 2004). Evidence suggests that OGIS has the highest validity and reliability, Matsuda provides the simplest equation to use, and both Gutt and Stumvoll allow for the inclusion of demographic variables in the equation (Hudak et al., 2021; Otten et al., 2014).

### 3.5 Fasting indices

#### 3.5.1 Theory and procedure

Fasting indices, shown in Table 2, can act as surrogate measures for both insulin sensitivity and β‐cell function (Otten et al., 2014). Two examples of common fasting indices are the homeostasis model assessment (HOMA) and the quantitative insulin‐sensitivity check index (QUICKI). Both HOMA and QUICKI are based on the feedback loop of insulin and glucose to maintain homeostasis (Katz et al., 2000; Wallace et al., 2004). During fasting, insulin levels and hepatic glucose production should remain constant (Katz et al., 2000; Wallace et al., 2004). When an individual is hyperglycaemic at fasting, insulin concentrations are insufficient to maintain effective glycaemic control. QUICKI can estimate insulin sensitivity, and the HOMA indices can estimate both insulin resistance (HOMA‐IR) and β‐cell function (HOMA‐β) (Katz et al., 2000; Wallace et al., 2004).

**TABLE 2:** Indices derived from fasting concentrations.

Indices	Equation
Quantitative insulin‐sensitivity check index (QUICKI) (Katz et al., 2000)	$=\frac{1}{\left[\log\left(\mathrm{Insuli}n_{0\;\min}\right)+\log\left(\mathrm{Glucos}e_{0\;\min}\right)\right]}$
Homeostasis model assessment for insulin resistance (HOMA‐IR) (Matthews et al., 1985)	$\;\frac{=(\mathrm{Insuli}n_{0\;\min}\;\times \;\mathrm{Glucos}e_{0\;\min})}{22.5}$
Homeostasis model assessment for β‐cell function (HOMA‐β) (Matthews et al., 1985)	$=(\frac{20\;\times \;\mathrm{Insuli}n_{0\;\min}}{\mathrm{Glucos}e_{0\;\min}-3.5})$

#### 3.5.2 Validity and reliability

The fasting indices can provide estimates of insulin sensitivity and β‐cell function based on the ability of glucose and insulin to maintain homeostasis (Muniyappa et al., 2008). During fasting conditions, the glucose concentration represents hepatic glucose production and the ability of insulin to stimulate the disposal of glucose produced endogenously (Muniyappa et al., 2008). Fasting insulin represents secretion from β‐cells, which will be higher or lower dependent on the insulin sensitivity of the individual (Muniyappa et al., 2008). When insulin secretion can no longer counteract impairments in insulin sensitivity, fasting hyperglycaemia prevails, evidenced in type 2 diabetes (Muniyappa et al., 2008). The indices therefore use the negative feedback loop between insulin and glucose to maintain euglycaemia (Muniyappa et al., 2008).

The relationship between insulin sensitivity derived from a hyperinsulinaemic euglycaemic clamp and fasting insulin sensitivity indices is hyperbolic, and logarithmic transformations of the indices are therefore recommended (Mather et al., 2001). The ability of both QUICKI and logHOMA‐IR to discriminate between individuals of differing insulin sensitivity, from lean to diabetic, is statistically comparable to the discriminant ratio of the hyperinsulinaemic euglycaemic clamp (Mather et al., 2001). QUICKI and logHOMA‐IR correlate well with the hyperinsulinaemic euglycaemic clamp in individuals with diabetes or obesity but correlate poorly in lean healthy subjects, suggesting that the indices perform worse in those who are insulin sensitive (Mather et al., 2001). QUICKI correlates well with the hyperinsulinaemic clamp to changes in insulin resistance owing to interventions, including diet and exercise, in individuals with type 2 diabetes (Katsuki et al., 2002). Correlation between repeated tests of logarithmically transformed indices has been assessed using Bland–Altman plots showing good test–retest reliability and uniform variability (Mather et al., 2001).

#### 3.5.3 Special considerations

HOMA and QUICKI are useful measures in epidemiological studies owing to the relatively low participant burden. Fasting indices fail to provide any indication of insulin sensitivity postprandially or in response to dynamic glucose or insulin concentrations. They are most useful in studies where other methods to measure insulin sensitivity are not feasible or where insulin sensitivity is a secondary research question. Care should also be taken when using the HOMA‐β index to measure β‐cell function because it should always be used in conjunction with a measure of insulin resistance (HOMA‐IR) (Matthews et al., 1985; Wallace et al., 2004).

## 4 SUMMARY

Glycosylated haemoglobin and CGMs provide an overall measurement of glycaemic control, particularly useful in clinical populations, but do not probe the physiology underlying glucose regulation, such as insulin sensitivity, glucose tolerance and β‐cell function. The hyperinsulinaemic euglycaemic clamp is the gold standard for measuring insulin sensitivity, and the hyperglycaemic clamp is the gold standard for measuring β‐cell sensitivity. Although highly standardised, both have a high participant burden and do not allow for dynamic measurements. The IVGTT allows glucose tolerance and an estimation of β‐cell function and insulin sensitivity to be measured with high reproducibility. Both the OGTT and the MMTT provide more dynamic measurements of glycaemic control and glucose tolerance but have poor reproducibility. The MMTT is most representative of daily life, but poor standardisation in the meal provides limited comparability between studies. The fasting indices are useful in epidemiological studies or in conjunction with other methods (Table 3).

**TABLE 3:** Methods to study glycaemic control and insulin sensitivity in human physiology research.

Method	Research recommendations	Important considerations
Glycosylated haemoglobin (HbA1c)	Measures glycaemia over the previous 120 days Often used clinically for diagnosis Useful for investigating intervention effects on glycaemic control Cannot measure acute glycaemic control or glycaemic variability	Do you have an individual trained in venepuncture? Do you have facilities to assess HbA1c concentration from the blood samples?
Continuous glucose monitor (CGM)	Measures free‐living glycaemia Can collect measurements for long periods Can measure glycaemic variability Low participant burden, most suitable for vulnerable populations Can be used as a useful secondary measure throughout different interventions	Have you followed the company training on how to fit the relevant CGM? Can you blind the device?
Oral glucose tolerance test (OGTT) and associated indices	Nutrition research Superior ecological validity Measure of glucose tolerance Estimates of insulin sensitivity from indices Useful for higher sample sizes because less equipment is required, and safer for patient groups than clamp methods	Do you have an individual trained to fit cannulas? Do you have a heated box or will you be using venous samples? Do you have equipment to measure glucose and insulin immediately or will this be done later? Do you have storage facilities for the blood samples (−70/80°C freezer)? Do you have immediate access to a refrigerated centrifuge to spin the blood samples?
Mixed meal tolerance test (MMTT)	Dynamic measurements of insulin sensitivity in response to nutritional intake Impact of proteins, fats and glucose on insulin sensitivity Measurements of β‐cell function taking into consideration incretin hormones Diurnal variations in insulin sensitivity	Do you have an individual trained to fit cannulas? Do you have a heated box or will you be using venous samples? Do you have equipment to measure glucose and insulin immediately or will this be done later? Do you have storage facilities for the blood samples (−70/80°C freezer)? Do you have immediate access to a refrigerated centrifuge to spin the blood samples?
Hyperinsulinaemic euglycaemic clamp	Gold standard for measuring insulin sensitivity Highly controlled research The main aim of the research is to investigate insulin sensitivity	Do you have an individual trained to fit cannulas? Do you have training on how to use the specialist equipment and a clinical member of staff to administer intravenous glucose/insulin and monitor the participant throughout? Do you have specialist training on using and storing isotopes? Radiolabelled isotopes Stable isotopes Will you be using an automated algorithm to calculate the glucose infusion rate during the experiment?
Hyperglycaemic clamp	Gold standard for measuring β‐cell function Highly controlled research Measures both first phase and second phase insulin secretory response Estimates whole‐body insulin sensitivity	Do you have an individual trained to fit cannulas? Do you have training on how to use the specialist equipment and a clinical member of staff to administer intravenous glucose/insulin and monitor the participant throughout? Do you have specialist training on using and storing isotopes? Radiolabelled isotopes Stable isotopes Will you be using an automated algorithm to calculate the glucose infusion rate during the experiment?
Intravenous glucose tolerance test (IVGTT)	A dynamic test of glucose tolerance, does not require steady‐state conditions Estimations of glucose effectiveness, insulin sensitivity and β‐cell secretion all from one test Useful to measure the acute insulin response after the glucose load	Do you have an individual trained to fit cannulas? Do you have training on how to use the specialist equipment and a clinical member of staff to administer glucose/insulin injection intravenously? Do you have specialist training on using and storing isotopes? Radiolabelled isotopes Stable isotopes Do you have an understanding of the mathematical modelling used to determine insulin sensitivity from this method?
Fasting indices	Large‐scale epidemiological studies Studies on high‐risk patients Studies on vulnerable populations Studies where only estimates of insulin sensitivity are required Studies where hepatic insulin resistance is to be estimated	Do you have an individual trained in venepuncture? Do you have facilities to assess glucose and insulin concentrations from the blood samples?

## AUTHOR CONTRIBUTIONS

All authors (Elizabeth Wrench, Daren Subar, Theodoros M. Bampouras, Robert M. Lauder and Christopher J. Gaffney) contributed to the conception and outline of the review. Elizabeth Wrench drafted the initial version, including figures, and all authors (Elizabeth Wrench, Daren Subar, Theodoros M. Bampouras, Robert M. Lauder and Christopher J. Gaffney) contributed to the final critical revision of this review. All authors have read and approved the final version of this manuscript and agree to be accountable and appropriately investigate any questions regarding the accuracy or integrity of any aspect of the work. All persons designated as authors qualify for authorship, and all those who qualify for authorship are listed.

## CONFLICT OF INTEREST

The authors have no conflicts of interest to report.
